# Coexistence of systemic sclerosis and cryopyrin‐associated periodic syndrome

**DOI:** 10.1111/1346-8138.17358

**Published:** 2024-06-28

**Authors:** Ai Kuzumi, Ayumi Yoshizaki, Kazuki Matsuda, Kojiro Nagai, Shinichi Sato

**Affiliations:** ^1^ Department of Dermatology University of Tokyo Graduate School of Medicine Tokyo Japan; ^2^ Department of Clinical Cannabinoid Research University of Tokyo Graduate School of Medicine Tokyo Japan

**Keywords:** autoantibody, autoimmune diseases, autoinflammatory diseases, cryopyrin‐associated periodic syndrome, systemic sclerosis

## Abstract

Systemic sclerosis (SSc) and cryopyrin‐associated periodic syndrome (CAPS) are distinct clinical entities belonging to the autoimmune and autoinflammatory diseases, respectively. The coexistence of the two entities has rarely been reported and is poorly characterized. Here, we described a case of a 38‐year‐old Japanese woman diagnosed with anti‐centromere antibody‐positive SSc and CAPS carrying the pathogenic mutation in the *NLRP3* gene, with a detailed autoantibody profile by a high‐throughput comprehensive protein array covering approximately 90% of the human transcriptome. The clinical manifestations of the patient were typical of both SSc and CAPS. Comprehensive autoantibody profiling identified 65 autoantibodies in the patient's serum and 78 autoantibodies in the serum of her daughter with CAPS, who carried the same *NLRP3* mutation as the patient. SSc‐associated autoantibodies (anti‐DBT, anti‐ CENP‐B, and anti‐CENP‐A) and anti‐CD320 antibody were detected at high levels only in the patient's serum, while autoantibodies to the following four proteins were detected in the sera of both the patient and her daughter: TRIM21, LIMS1, CLIP4, and KAT2A. The TRRUST enrichment analysis identified NF‐κB1 and RelA as overlapping key transcription factors that regulate the genes encoding proteins to which autoantibodies were detected in the patient and her daughter, therefore the autoantibody profile of the patient cannot be solely attributed to SSc, but may also be influenced by CAPS. Although autoimmune and autoinflammatory diseases are considered to be at opposite ends of the immunological spectrum, detailed autoantibody profiling may reveal a unique immunological landscape in an overlapping case of the two entities.

## 1 INTRODUCTION

Systemic sclerosis (SSc) is an autoimmune disease characterized by vasculopathy and fibrosis of the skin and internal organs that affects approximately 2.5 million people worldwide. Although the pathogenesis of SSc is not fully understood, accumulating evidence points to a complex interplay between immune dysregulation, endothelial dysfunction, and fibroblast activation in the disease.^1^ Cryopyrin‐associated periodic syndrome (CAPS) is a rare autoinflammatory disease with an estimated prevalence of one to two cases per million. CAPS is caused by gain‐of‐function mutations in the *NLRP3* gene, resulting in aberrant activation of the NLRP3 inflammasome and subsequent overproduction of interleukin (IL)‐1β. Clinical features of CAPS include rash, fever, arthritis, conjunctivitis, chronic meningitis, and sensorineural hearing loss.^2^

Autoimmune and autoinflammatory diseases are considered distinct clinical entities resulting from dysregulation of the adaptive and innate immune systems, respectively.^3^ The coexistence of these two entities has rarely been reported. Here, we describe a unique case of SSc and CAPS occurring in the same patient.

## 2 CASE REPORT

A 38‐year‐old Japanese woman was referred to our department with a chronic non‐pruritic urticarial rash that occurred almost every day since the age of 6 months. She had been treated with antihistamines and the anti‐IgE antibody omalizumab without improvement. In addition, she had had periodic fever attacks once a month throughout the year since childhood that lasted 3–4 days and were accompanied by chills, malaise, and joint pain. The urticarial rash occurred almost daily, regardless of fever. In the past few years, she had suffered from Raynaud's phenomenon. Her medical history was otherwise unremarkable. Her 2‐year‐old daughter developed a similar rash every day from the second day after birth. The daughter also had periodic fevers once a month, each lasting 3–4 days. There was no other relevant family history.

**FIGURE 1:**
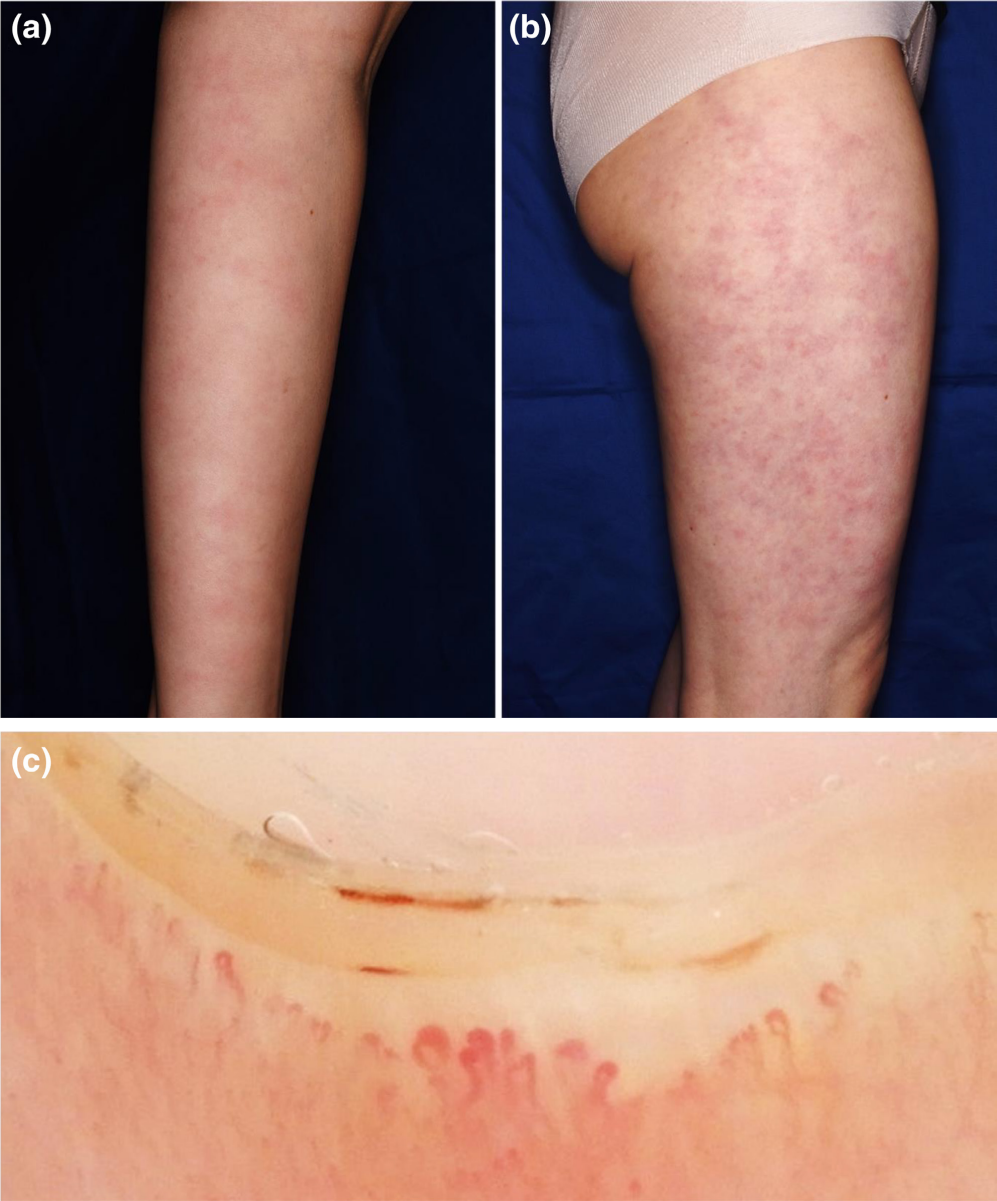
Family pedigree and clinical findings. (a, b) Edematous erythema on the extremities. (c) Enlarged capillaries at the nailfold.

**FIGURE 2:**
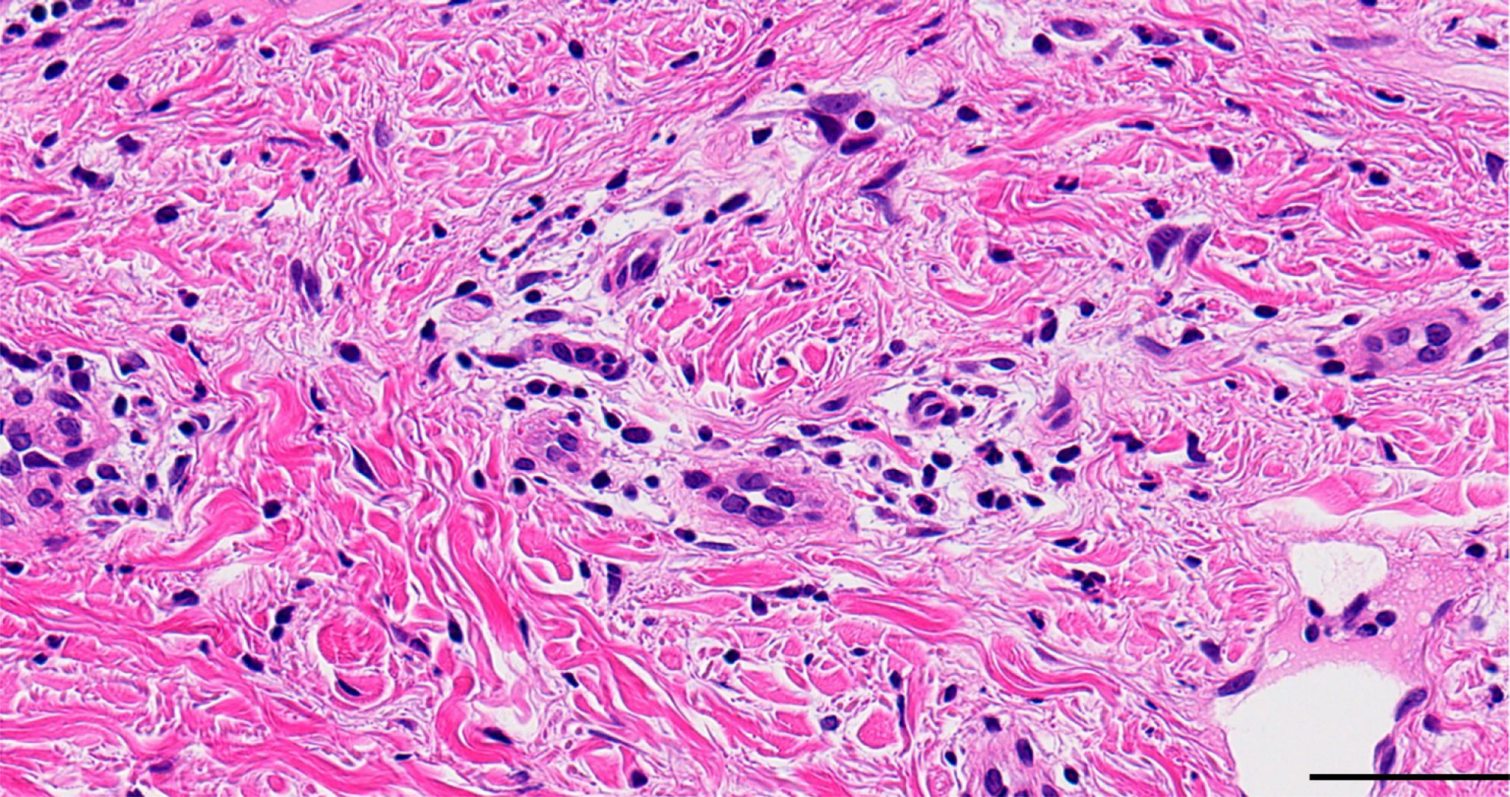
Histopathological findings. Histopathology showing perivascular infiltration of lymphocytes and neutrophils in the dermis with diffuse dermal neutrophilia (hematoxylin–eosin stain, original magnification ×400). Scale bar = 50 μm.

Physical examination of the patient revealed edematous erythema of the extremities (Figure 1a,b). She also had puffy fingers, and enlarged capillaries were observed at the nailfold (Figure 1c). Laboratory investigations showed elevated C‐reactive protein (4.54 mg/dL, normal <0.30 mg/dL), erythrocyte sedimentation rate (44 mm/h, normal 3–15 mm/h), and amyloid A (53.1 mg/L, normal <3.0 mg/L). Serum D‐dimer level was within normal limits (0.5 μg/mL, normal <1.0 μg/mL). Serological tests were positive for antinuclear antibody (1:2560, discrete speckled), anti‐centromere antibody (763 U/mL, normal <10.0 U/mL), and anti‐mitochondrial M2 antibody (400 U/mL, normal <10.0 U/mL). Anti‐SS‐A antibody was negative. She had no complications of Sjogren's syndrome. Pulmonary function tests were within normal limits. Echocardiography showed no evidence of pulmonary artery hypertension. Hearing tests were normal. Skin biopsy from an edematous erythema of the left arm showed perivascular infiltration of lymphocytes and neutrophils in the dermis with diffuse dermal neutrophilia (Figure 2), compatible with CAPS.^4^ After obtaining informed consent, genetic testing was performed and revealed a heterozygous missense mutation (NM_004895.4: c.1322C>T, p.Ala441Val) in the *NLRP3* gene, previously reported as a pathogenic variant for CAPS.^5^ The diagnosis of CAPS was made. Specifically, the chronic/intermittent episodes of fever, urticarial rash, and arthralgia in the absence of a specific trigger in our patient led to the diagnosis of Muckle–Wells syndrome, an intermediate phenotype of CAPS.^5^, ^6^, ^7^ She also met the 2013 ACR/EULAR criteria for SSc (puffy finger, Raynaud's phenomenon, anti‐centromere antibody, and abnormal nailfold capillaries).^8^ The modified Rodnan skin thickness score^9^ was 4. Treatment of CAPS was started with the anti‐IL‐1β monoclonal antibody canakinumab (150 mg stands for subcutaneously (s.c.) every 8 weeks). After starting canakinumab, she had no episodes of rash or fever. Serum C‐reactive protein levels and erythrocyte sedimentation rate decreased to the normal range. The manifestations of SSc, including nailfold vascular abnormalities, remained unchanged. Her daughter was diagnosed with CAPS with the same *NLRP3* mutation and successfully treated with canakinumab (4 mg/kg s.c. every 8 weeks).

Given the rare coexistence of SSc and CAPS, the patient's autoantibody profile was further examined by a high‐throughput comprehensive protein array covering approximately 90% of the human transcriptome, as previously described.^10^, ^11^, ^12^ The autoantibody profile of her daughter, who had only CAPS and was negative for antinuclear antibody, was also studied.

**FIGURE 3:**
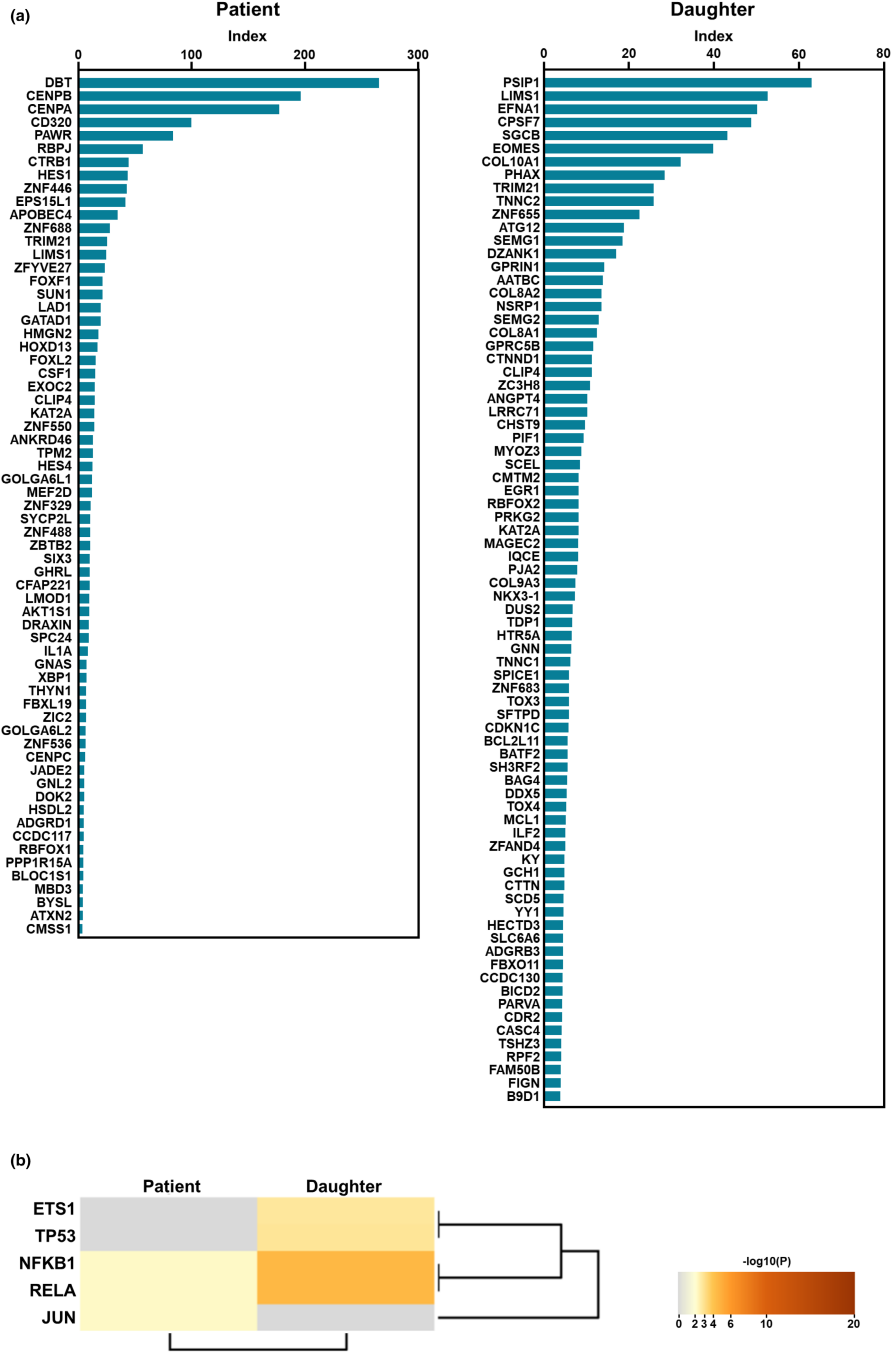
Autoantibody profile of the patient and her daughter. (a) Autoantibodies detected in the patient and her daughter by a high‐throughput multiplex protein array covering approximately 90% of the human transcriptome. (b) TRRUST enrichment analysis showing NF‐κB1 and RelA as overlapped key transcription factors that regulate the genes encoding proteins to which autoantibodies were detected in the patient and her daughter.

A total of 65 and 78 autoantibodies were detected in the sera of the patient and her daughter, respectively (Figure 3a). Autoantibodies to DBT, CENP‐B, CENP‐A, and CD320 were detected at high levels only in the patient's serum, the first three of which were consistent with the positivity for anti‐centromere and anti‐mitochondrial M2 antibodies. Autoantibodies to the following four proteins were detected in the sera of both the patient and her daughter at relatively low levels: TRIM21, LIMS1, CLIP4, and KAT2A. Moreover, TRRUST enrichment analysis^13^, ^14^ identified NF‐κB1 and RelA as overlapping key transcription factors that regulate the genes encoding proteins to which autoantibodies were detected in the patient and her daughter (Figure 3b).

## 3 DISCUSSION

To the best of our knowledge, this is the first case of coexistence of SSc and CAPS. Puffy fingers, Raynaud's phenomenon, abnormal nailfold capillaries, and anti‐mitochondrial M2 antibody in the patient were typical of anti‐centromere antibody‐positive SSc.^1^ Regarding the clinical manifestations of CAPS, a study by Levy and colleagues shows that the Ala441Val variant is associated with a mild phenotype with a low risk of hearing loss and neurological involvement,^5^ as was the case with the patient that we described. Therefore, the phenotypes of SSc and CAPS did not seem to affect each other in the patient, consistent with the distinct pathogeneses of autoimmune and autoinflammatory diseases. This is also supported by the unchanged clinical manifestations of SSc after canakinumab therapy.

On the other hand, comprehensive autoantibody profiling revealed both unique and overlapping patterns of autoantibodies in the patient and her daughter. Autoantibodies detected exclusively in the patient but not associated with SSc include anti‐CD320 antibody, which has been reported in cutaneous arteritis.^10^ Although the significance of anti‐CD320 autoantibody in the patient is unclear, vascular and perivascular inflammation caused by SSc and CAPS might lead to the release of CD320 into the circulation, resulting in the production of anti‐CD320 antibody, as CD320 is highly expressed in vascular endothelial cells.^10^

The patient and her daughter shared several autoantibodies, although autoinflammatory diseases, including CAPS, are not generally associated with autoantibody production. Indeed, most of the autoantibodies detected in the patient and her daughter have no known clinical significance in CAPS, including those against TRIM21, LIMS1, CLIP4, and KAT2A, which were detected in both. However, considering that the number of autoantibodies can serve as a marker of inflammation in various diseases,^12^ the large number of autoantibodies detected in the patient and her daughter may reflect the inflammatory status in their bodies. Moreover, NF‐κB1 and RelA were identified as overlapping key transcription factors regulating the genes encoding proteins to which autoantibodies were detected in the patient and her daughter. Since these transcription factors are important for the activation of NF‐κB, which is essential for IL‐1β production by the NLRP3 inflammasome in CAPS,^15^ the autoantibodies detected in the patient and her daughter may be relevant in CAPS and should be explored as potential biomarkers of the disease.

In summary, we described a unique case of coexistence of SSc and CAPS with a detailed autoantibody profile. The clinical manifestations were typical of both diseases, while diverse autoantibodies were detected that cannot be attributed solely to SSc, suggesting the influence of CAPS on autoantibody production. Although autoimmune and autoinflammatory diseases are considered to be at opposite ends of the immunological spectrum, comprehensive autoantibody detection may reveal a unique immunological landscape in an overlapping case of the two entities, bridging the gap between autoimmunity and autoinflammation.

## CONFLICT OF INTEREST STATEMENT

A.Y. belongs to the Social Cooperation Program, Department of Clinical Cannabinoid Research, supported by Japan Cosmetic Association and Japan Federation of Medium & Small Enterprise Organizations. Other authors have declared that no conflict of interest exists.

## INFORMED CONSENT

Written informed consent was obtained from the patient.
